# Prenatal and maternal study of halloysite toxicity in pregnant rats

**DOI:** 10.1038/s41598-025-20686-3

**Published:** 2025-10-01

**Authors:** Ahlam G. Khalifa, Asmaa K. Abdelghany, Emad A. Mahdi, HebatAllah H. Mahmoud, Nour El-Houda Y. Hassan

**Affiliations:** 1 Department of Forensic Medicine and Toxicology, Faculty of Veterinary Medicine, Beni-Suef University, Beni-Suef, 62511 Egypt; 2 Animal and Poultry Management and Wealth Development Department, Faculty of Veterinary Medicine, Beni-Suef University, Beni-Suef, 62511 Egypt; 3 Pathology Department, Faculty of Veterinary Medicine, Beni Suef University, Egypt, Egypt; 4 Anatomy and Embryology Department, Faculty of Veterinary Medicine, Beni-Suef University, Beni-Suef, Egypt; 5 Department of Forensic Medicine and Toxicology, Faculty of Veterinary Medicine, Beni-Suef University, Beni-Suef, 62511 Egypt

**Keywords:** Halloysite nanoclay, Intranasal, Gestation, Litter size, Oxidative stress, Environmental sciences, Materials science

## Abstract

Halloysite nanoclay (HNC) is a naturally occurring tubular aluminosilicate that has various applications in nanotechnology and drug delivery. However, its toxic effects during gestation are inadequately reported. This study assessed the maternal and fetal toxic effects of intranasally and orally administered HNC. Pregnant rats were divided into three groups: vehicle control, oral HNC (75 mg/kg), and intranasal HNC at the same dose from day 0 to day 19 of gestation (day after day). Dams showed weight loss, but HNC did not cause lethality in both groups. HNC reported route-dependent tissue toxicity. Orally administered HNC leads to more pronounced oxidative intestinal damage than intranasal treatment. Intranasal administration has a much greater impact on reducing thiol content in the lungs compared to oral administration. Histopathological analysis revealed that the fetal pancreas of dams treated with HNC intranasally showed marked necrotic acini and congested blood vessels, whereas the dams treated orally exhibited foamy macrophages and lung angiopathy more frequently than the intranasally treated ones. These results highlighted that HNC, at the tested dose, caused significant pathological and oxidative damage to maternal and fetal tissues.

## Introduction

Nanoclay property and application research is a rapidly growing field, driven by its significant potential in biomedical applications^1^. Halloysite nanotubes (HNTs) are naturally occurring, hollow, cylindrical nanomaterials derived from the kaolin clay group^2^. Their unique structure, high surface area, and biocompatibility have led to several applications, including drug delivery, catalysis, environmental rehabilitation, and reinforcement in composite materials. HNTs have also shown promise in biomedical applications due to their ability to encapsulate and deliver therapeutic agents^3^.

The integration of polymeric substances like polycaprolactone, polyethyleneimine, poly(N-isopropyl acrylamide), endodontic sealers, gelatin elastomers, chitosan, or adhesive resins^4^ to HNT functionality provides additional properties that include antimicrobial properties^5^, cancer-fighting activity^6^, therapy for neurological diseases^7^, substances for healing wounds, bone restoring^8^, regeneration of tissues^9^, and filler material for broad-application substances^10^. However, like other nanomaterials, concerns have been raised regarding the potential toxicity of HNTs. While generally considered biocompatible, the effects of HNTs on various biological systems, especially with long-term exposure, require careful investigation^11^.

Workers who create, handle, or use HNTs are exposed to them at work, primarily by inhalation or skin contact. Additionally, ingesting or coming into contact with consumer goods that contain HNTs can expose on self to them. Oral administration in mice has been linked to pulmonary fibrosis and liver oxidative damage^12^. Furthermore, increased HNT surface area has been shown to exacerbate pulmonary inflammation in mice. Functionalization also plays a critical role in toxicity, as demonstrated by the induction of apoptosis in rat glioblastoma cells by HNTs functionalized with specific silanes^13^.

Because HNTs and carbon nanotubes (CNTs) have comparable tubular structures, they may be equally harmful. This toxicity could involve malignant alterations in the human respiratory system, pulmonary inflammation, and an acute phase reaction post-pulmonary exposure^14,15^Considering the numerous applications of HNTs and their nanoscale dimensions (30–70 nm in diameter and 1 to 3 μm in length), evaluating the maternal-fetal interface of HNTs through the placental barrier is crucial for their safety.

The placental barrier has been described as “a lipid membrane that permits bidirectional transfer of substances between maternal and fetal compartments.” The majority of pesticides, plant alkaloids, mycotoxins, metals, and other xenobiotics enter the placenta easily, as does any compound with a molecular weight (MW) less than 1,000^16^.

There is little evidence available regarding the toxicological effects of HNTs on human health^17^. Consequently, there is a growing need to look into the possible dangers and effects of HNC. Addressing this gap is essential to developing safer HNC-based products and ensuring responsible handling practices, thereby protecting human health and facilitating the continued innovation of these promising nanomaterials for public benefit. (Especially relevant for drug delivery, tissue regeneration, etc.). Thus, after exposing pregnant rats to HNC via multiple ways of administration, particularly on first-generation offspring, the current research evaluated the toxic effects of HNC.

## Results

### High-resolution transmission electron microscope (HRTEM)

**Fig. 1:**
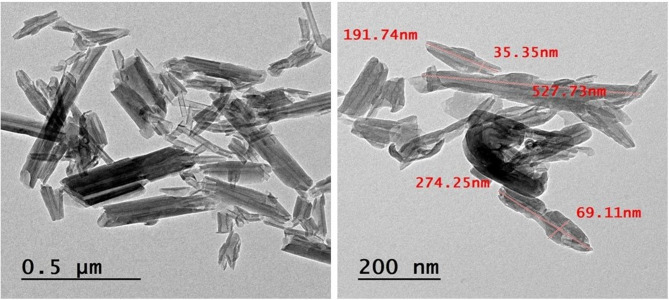
TEM micrograph of HNTs.

The HRTEM image displayed HNT nanoparticles as tube- or rod-shaped structures with a length of 0.5 μm and a diameter of 35 nm (Fig. 1).

### The effect of halloysite administration on the weight of pregnant rats and litter size, as well as its clinical signs of toxicity

The toxic effects and lethality of HNC administered to pregnant rats via oral and intranasal administration at a dose of 75 mg/kg produced loss of weight in both the oral-treated halloysite group (HO) and the intranasal-treated halloysite group (HI) treated females, while vaginal and urethral bleeding was observed in some females in the HI group at the end of the 1 st week of pregnancy, and some resorbed sites were observed in the HO group at the time of euthanasia (20 days). HNC did not cause lethality in both groups (Table 1).

**Table 1:** Toxicity of Halloysite in pregnant rats (clinical signs).

Halloysite-treated pregnant rats	Number of pregnant rats	Route of administration	Exposure period	Toxic effects	Lethality %
**HI**	**10**	**Intra nasal**	**19 days**	**-Vaginal**,** urethral bleeding** **- Weight loss** **-Abortion**	**0**
**HO**	**10**	**Oral**	**19 days**	**-Resorption of some fetuses** **- Weight loss**	**0**

HI: Intranasal-treated halloysite dams’ group.

HO: Oral-treated halloysite dams’ group.

**Fig. 2:**
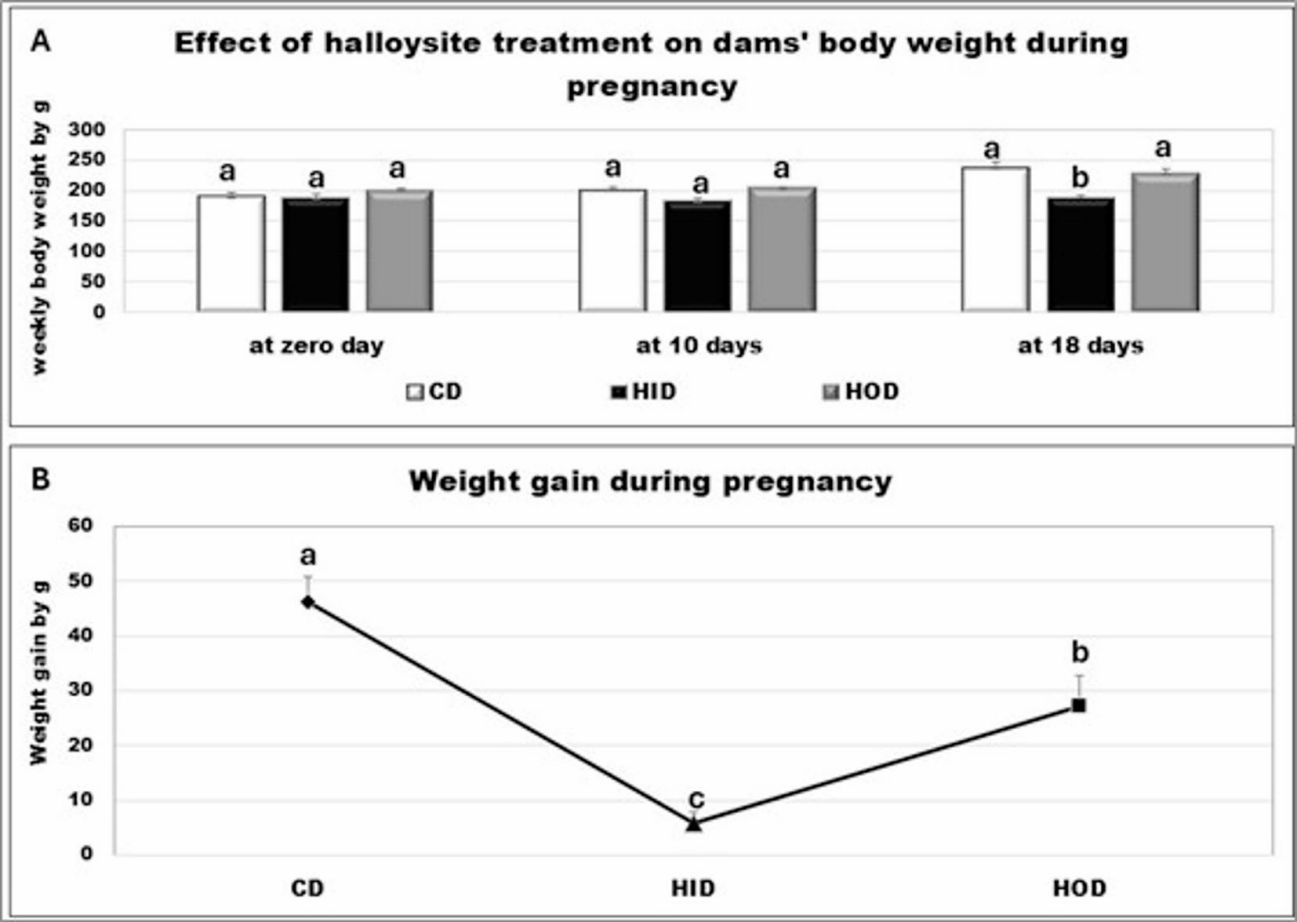
Effect of treatment with halloysite on dams’ body weight and weight gain during pregnancy. Results expressed as mean ± SE performed by one-way ANOVA followed by post hoc Tukey test. a, b, & c letters indicate significant difference at *P* < 0.05. C: Control dams’ group. HI: Intranasal-treated halloysite dams’ group. HO: Oral-treated halloysite dams’ group.

The increase in body weight under the influence of HNC during pregnancy was remarkably (*P* < 0.05) higher in the control dams and oral-treated halloysite dams (HO) groups than in the intranasal-treated halloysite dams (HI) group at 18 days of pregnancy. On the other side, both the HI and HO groups showed an evident reduction in weight gain during pregnancy with respect to the control group, while the HI group showed the most prominent reduction (Fig. 2).

**Fig. 3:**
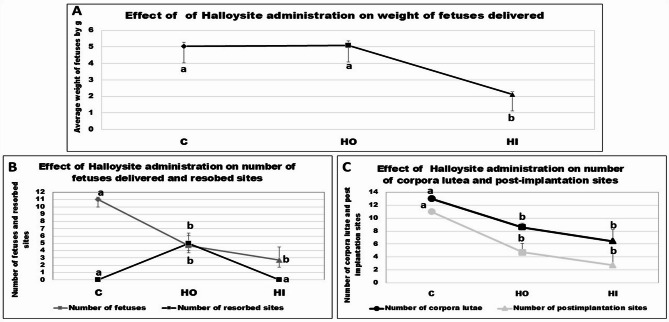
Effect of administration of halloysite to pregnant rats during days 0–19 on fetuses delivered on day 20 of pregnancy. Results expressed as mean ± SE performed by one-way ANOVA followed by post hoc Tukey test. a, & b letters indicate a significant difference at *P* < 0.05, while similar letters indicate no significance. C: Control fetuses’ group. HI: Intranasal-treated dams halloysite fetuses’ group. HO: Oral-treated dams halloysite fetuses’ group.

The fetal weight of litters delivered at 20 days of pregnancy was not significantly decreased in the HO group; however, a marked (*P* < 0.05) reduction was noted in the HI group. The litter size (number of fetuses) was markedly (*P* < 0.05) reduced in the halloysite-treated groups in relation to the control dams. It was evident that the oral halloysite administration raised the number of resorbed fetuses with respect to the control and intranasal-treated halloysite (HI) group. The number of corpora lutea and post-implantation sites was significantly (*P* < 0.05) reduced in the halloysite-treated groups with respect to the control group (Fig. 3).

### Effects of halloysite administration on insulin levels in pregnant rats

It was evident that the HO group had elevated insulin levels with respect to the control group, but this elevation failed to attain statistical significance. Conversely, the HI group showed a decrease in insulin level than the control group, and the HO group had a slightly higher insulin level compared to the HI group. This suggests that oral treatment increased insulin levels relative to intranasal (Table 2).

**Table 2:** Effects of Halloysite administration on insulin levels in pregnant rats.

Groups	Control	HI	HO
parameters			
**Insulin(µU/mL)**	**0.47 ± 0 0.035** ^**ab**^	**0.40 ± 0 0.003** ^**b**^	**0.56 ± 0.044** ^**a**^

Results expressed as mean ± SE were performed by one-way ANOVA followed by post hoc Tukey test.

^a & b^ superscripts in the same row indicate a significant difference at *P* < 0.05, while similar letters indicate no significance.

Control: Control dams’ group.

HOD: Oral-treated halloysite dams’ group.

HID: Intranasal-treated halloysite dams’ group.

### Effects of halloysite on antioxidant markers (total thiols, glutathione) and malondialdehyde (MDA) content in the pregnant rats’ lung and intestinal tissues

Concerning oxidative damage in the lungs, the HI group revealed a marked reduction in total thiol content in relation to both control and HO groups, and a marked increase in MDA content. In contrast, both the HI and HO groups exhibited a significant reduction in lung glutathione (GSH) content than the control group. In the intestinal tissue, both HO and HI groups showed a marked decrease in thiol levels compared to the control dams. However, the GSH level was markedly reduced in the HO dams compared to the control. A marked elevation in MDA content was observed in the HO group in relation to both the HO and control groups (Table 3).

**Table 3:** Effects of Halloysite on antioxidant markers (nmol/100 mg tissue) and lipid peroxidation (nmol MDA/100 mg Tissue/Hour) in the lung and intestinal tissues of pregnant rats.

Groups	Control	HI	HO
parameters			
lung tissue			
Total thiols	**534.31 ± 9.80** ^**a**^	**230.39 ± 4.31** ^**b**^	**539.21 ± 4.90** ^**a**^
GSH	**21.69 ± 1.35** ^**a**^	**16.26 ± 1.05** ^**b**^	**16.26 ± 1.05** ^**b**^
Lipid Peroxidation (MDA)	**1.11 ± 0.15** ^**a**^	**6.82 ± 0.73** ^**b**^	**4.76 ± 0.23** ^**ab**^
Intestinal tissue			
Total Thiols	**90.19 ± 7.70** ^**a**^	**39.21 ± 4.90** ^**b**^	**36.27 ± 6.86** ^**b**^
GSH	**24.67 ± 2.98** ^**a**^	**21.28 ± 1.50** ^**ab**^	**17.62 ± 0.54** ^**b**^
Lipid Peroxidation (MDA)	**0.66 ± 0.11** ^**a**^	**2.26 ± 1.05** ^**a**^	**4.90 ± 1.85** ^**b**^

Results expressed as mean ± SE were performed by one-way ANOVA followed by post hoc Tukey test.

^a & b^ superscripts in the same row indicate a significant difference at *P* < 0.05, while similar letters indicate no significance.

Control: Control dams’ group.

HO: Oral-treated halloysite dams’ group.

HI: Intranasal-treated halloysite dams’ group.

### Gross anatomical and X-ray examination of fetuses

**Fig. 4:**
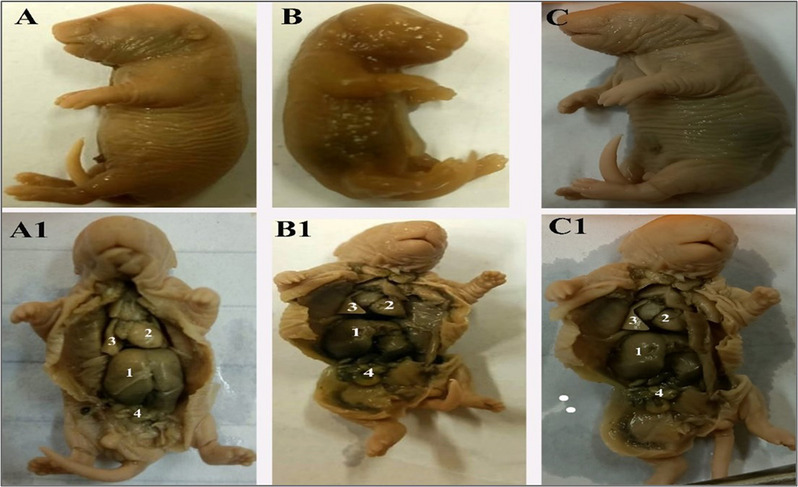
**A**- fetus from the control group, **B-** fetus from the dam treated with halloysite intranasally, showing darkness of skin, **C**- fetus from the dam treated with halloysite orally, showing edema. A1, B1, C1- showing internal organs of fetus in three groups (1-liver,2-heart,3-lung,4-intestine).

**Fig. 5:**
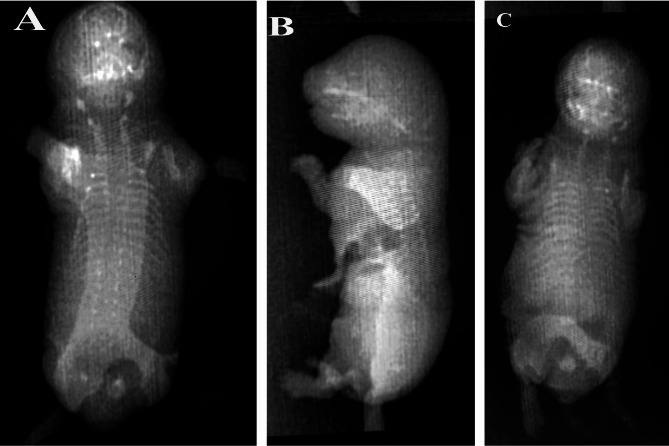
X-ray imaging: fetus from control group **(A)** is normal, fetus from HI-treated dams, showing incomplete vertebral column with abnormalities in ribs **(B**), fetus from Ho-treated dams showing darkness of skin **(C**).

Observation of the fetal skeletons was done for a quarter of the live fetuses, which were examined grossly by using a magnifying glass (Fig. 4**)** and X-ray examination in Fig. 5. Skeletal evaluations in HI-treated dams showed incomplete vertebral columns with abnormalities in ribs (deviation and delayed ossification;10%), while in HO dams, they showed incomplete ossification and darkness of skin (10%).

The X-ray view of the control fetal skeleton (Fig. 5, A) revealed that the skull is ossified and proportionate in shape, with no gross deformities. spine appeared straight, with vertebrae aligned. Ribs appear bilaterally symmetrically with no rib fusion or missing ribs. As well as the limbs were spread out to the sides. The tail was visible.

The X-ray view of the HI fetal skeleton (Fig. 5, B) showed that the body shape is preserved. The skull is relatively round. The facial area appears somewhat flattened. The vertebral column showed an incomplete vertebral column with impaired ossification in the ribs. Uneven space between ribs. Also, some ribs were closer together, and others were more widely spaced. Limbs were visible with reduced clarity of bone margins, and pelvic bones were not well visualized.

The X-ray view of the HO fetal skeleton (Fig. 5, C) showed a rounded skull and bilaterally distributed ribs. Jaw bones do not delineate. Thoracolumbar vertebrae appear faint. Ribs were irregular in spacing. Pelvic bones were poorly visualized. Limbs showed incomplete ossification of the long bones.

### Histopathological study

#### Microscopic evaluation of rat and fetal lung tissues

**Fig. 6:**
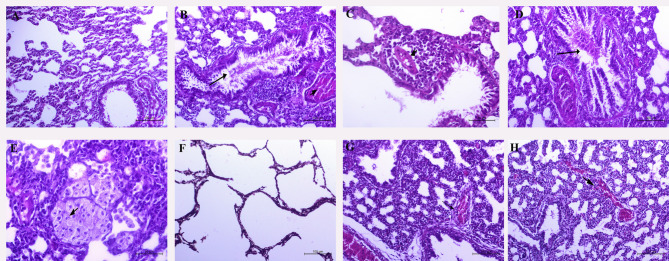
Representative photomicrographs of rat lung sections stained with Hematoxylin–Eosin stain (magnifications 200x and 400X): (**A**) normal control rats (group **A**) revealed the normal histological structure of pulmonary tissue. Rats intranasal treated with halloysite (**B** and **C**) showed marked congestion (head arrow), and edema (arrow) with massive perivascular and peribronchiolar mononuclear cells cuffing. Rats orally treated with halloysite (**D** and **E**). The bronchial epithelium revealed hyperplastic, dysplastic, and squamous metaplastic changes and its lumen was partially obliterated with desquamated and folded surface epithelial lining(arrow) (**D)**. (**E**) Characteristic pale foamy macrophages were free lying or packed in alveoli (head arrow). (**F**) fetal lungs of normal control rats showed normal histologic structure of alveolar walls and bronchi, while lungs of both groups of rats treated with halloysite intranasally(**G**), and those rats treated with halloysite orally (**H**) showed marked congestion (arrow), edema, hemorrhages (head arrow), and bronchitis.

Figure (6), H and E-stained lung sections of normal control rats (Fig. 6A) revealed the normal histological structure of pulmonary alveoli, alveolar walls, and bronchioles. Most of the pulmonary sections of the HI group showed marked congestion and edema with massive perivascular and peribronchiolar mononuclear cell cuffing. Angiopathy was severe, and in some cases, the vascular wall was necrosed and replaced with acidophilic, structureless coagulum within a thick mononuclear cuff. The bronchial epithelium revealed hyperplastic, dysplastic, and squamous metaplastic changes, and its lumen was partially obliterated with desquamated and folded surface epithelial lining. Characteristic large, pale, foamy macrophages were detected within the pulmonary alveoli. These macrophages could be found living freely or enclosed in alveoli with granular extracellular material. Fine eosinophilic granules were occasionally seen in their vacuolated, copious cytoplasm (Fig. 6B and C), and “HO” groups presented all the previously mentioned pulmonary pathology, but in a less severe degree in some cases (Fig. 6D and E).

The fetal lungs of control group fetuses showed normal histologic structure of alveolar walls and bronchi (Figure F), while lungs of both HI and HO groups of fetuses showed marked congestion, edema, hemorrhages, and bronchitis (Fig. 6G and H).

#### Microscopic evaluation of rat and fetal intestinal tissue

**Fig. 7:**
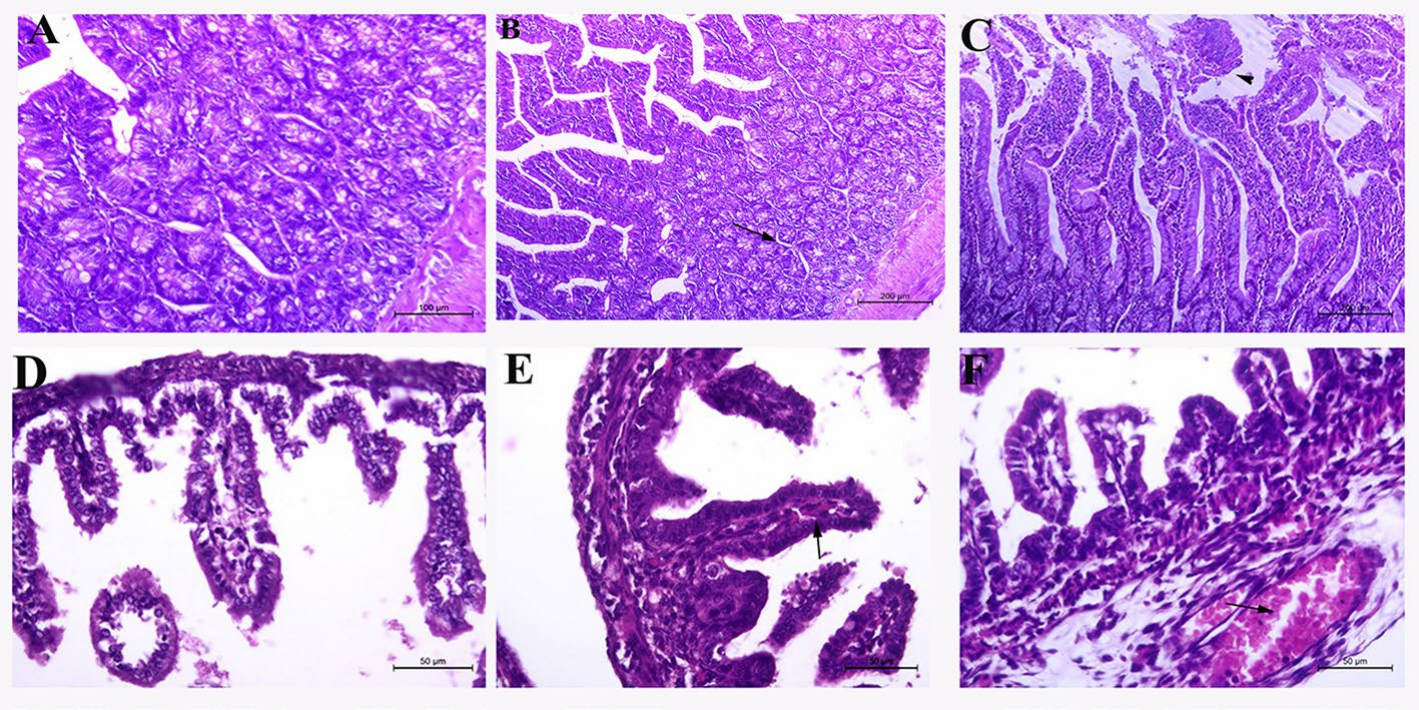
Representative photomicrographs of rat small intestinal sections stained with Hematoxylin–Eosin stain (magnifications 100x,200x, and 400X): (**A**) Normal control rats (group A) revealed the normal histological structure of mucosa, submucosa, and musculosa. intestinal sections of rats intranasally treated with halloysite (**B**) revealed mild edema (arrow) in comparison to rats orally treated(**C**) which presented nectobiotic and desquamative mucosal changes(head arrow) with submucosal mononuclear cell infiltration.(**D**) The fetal intestinal segments of normal control rats showed the normal histological structure of mucosa, submucosa, and musculosa, while the intestine of both groups of rats treated with halloysite intranasally(**E**), and those rats treated with halloysite orally(**F**) showed mild to moderate congestion(arrow), and edema, respectively.

The stained small intestine sections of normal pregnant rats (Fig. 7A) revealed the normal histological structure of mucosa, submucosa, and musculosa. Most of the intestinal sections of rats intranasally treated with halloysite (Fig. 7B) revealed mild edema in comparison to rats orally treated, which presented necrobiotic and desquamative mucosal changes with submucosal mononuclear cell infiltration (Fig. 7C).

The fetal intestinal segments of normal control rats showed the normal histological structure of mucosa, submucosa, and musculosa, while the intestine of both groups of HI and HO showed mild to moderate congestion and edema, respectively (Fig. 7D and E, and 7F).

#### Microscopic evaluation of fetal pancreas

**Fig. 8:**
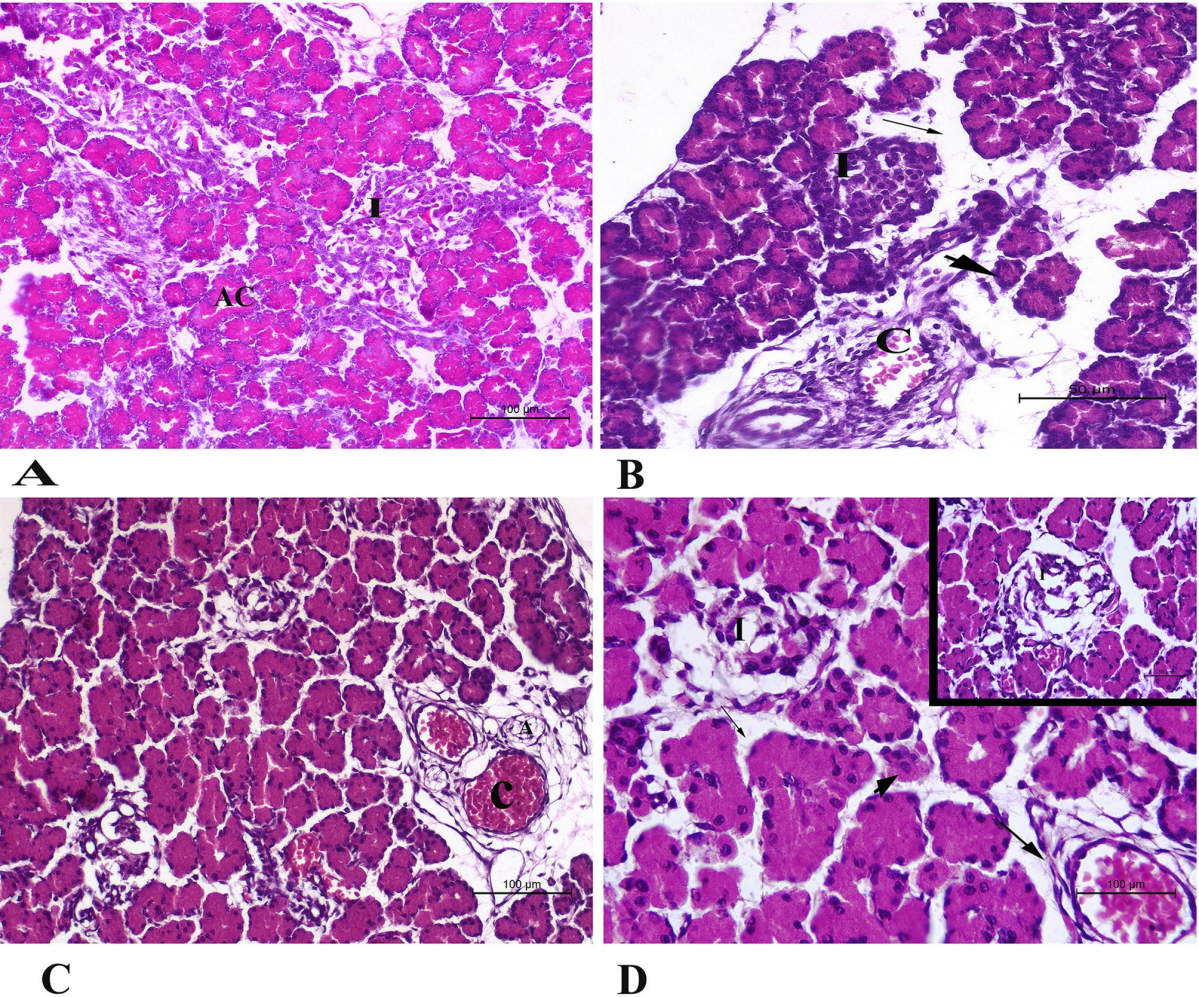
Representative photomicrographs of rat fetal pancreas sections stained with Hematoxylin–Eosin stain (magnifications 100x and 50X): **(A**) normal control fetal pancreas (group A) revealed normal islets of Langerhans(I)with normal acinar arrangements (AC). The fetal pancreas of both groups of rats treated with halloysite intranasally **(B)**, and those rats treated with halloysite orally **(C** and **D)** showed destroyed islets of Langerhans (I), congestion of the blood vessels(C), and atrophied blood vessels (A). The acini were destroyed (Head of arrow) and surrounded by oedema (thin arrow). Also, there was peri-vascular oedema (Thick arrow).

The fetal pancreas of normal control rats showed normal histologic structure of islets of Langerhans, which are scattered throughout the pancreas. These islets are distinct from the surrounding tissue and appear lightly stained compared to the darker exocrine tissue. The acini are arranged in lobules, separated by thin layers of connective tissue that contain blood vessels. The fetal pancreas of HO rats showed marked congestion of the blood vessels. The tunica intima appears dilated with endothelium detachment. Surrounding the congested blood vessels, there is an accumulation of fluid in the interstitial spaces, seen as pale or clear areas around the vessels. Similar fluid accumulation is observed around the acinar cells. The cells within the islets appear necrotic, or destroyed, with a loss of normal cellular organization. Some secretory acini showed necrotic and atrophied changes. The HI group presented all the previously mentioned pancreatic pathology, but to a less severe degree in some cases (Fig. 8).

## Discussion

Halloysite nanotubes (HNTs) hold great promise in biomedicine, including the creation of tissue engineering bone implantation, ultrasound contrast agents, tooth fillings, drug and gene transfer vesicles, tumor and stem cell isolation, and cosmetics^18^. Considering the many applications of HNTs, evaluating their toxicity is essential for the safety of workers. There is little data available regarding the toxicological impact of HNTs on human health^1^. Generally, a decrease in weight gain in treated groups in comparison with the control is regarded as a negative outcome when evaluating toxicity^19^Moreover, the fetal weight, embryo abnormalities, urethral bleeding, and fetal resorption are the most prominent measures evaluated in the toxicity studies^20^.

Nanomaterials have become increasingly prevalent in various sectors, from industry and medicine to everyday consumer products. While these materials offer numerous interactions with biological systems, along with potential consequences raise concerns about unpredictable risks to human health. Halloysite, a naturally occurring nanosized tubular clay mineral, stands out among nanomaterials due to its diverse applications. It finds utility in fields such as environmental science, biomedicine, wastewater treatment, cosmetics, nanoelectronics, dye removal, nanocomposite fabrication, and even forensic science. Despite its potential, halloysite, like other nanomaterials, necessitates careful consideration of its potential impact on human health and the environment^8^. Our study investigates the potential toxic effects of halloysite nanotubes (HNTs) on pregnant rats and their offspring. Specifically, the research aims to assess HNT-induced toxicity in the maternal intestine and pancreas and to evaluate the impact on fetal development.

Nanoparticles have been shown to increase mitochondrial reactive oxygen species (ROS) levels, which results in mitochondrial malfunction^21,22^. Determining the total oxidative/total antioxidative condition is crucial in toxicology studies since ROS generate oxidative stress in cells. Changes in cell motility, apoptosis, cytotoxicity, uncontrolled cell signaling, DNA damage, and the onset of cancer are all caused by an excess of ROS^23^. In our study, we evaluated MDA, glutathione content, and total thiol levels in lung and intestinal tissue homogenates of pregnant rats after oral and intranasal exposure to 75 mg/kg 3 times per week. In the treated group of intranasal exposure, significant changes in evaluated glutathione content and total thiol levels compared with HO were observed. Our findings are consistent with those reported by Wang et al.^12^ and Hu et al.^24^ that HNTs induced significant oxidative damage in the lung and small intestine after thirty days of oral gavage in mice, especially at the high dose (50 mg/kg), where the MDA level was elevated and glutathione levels were reduced. HCN showed localized lung effects due to initial deposition in the nasal cavity/lungs. HCN may disrupt alveolar redox balance by interacting with alveolar macrophages or lung surfactant proteins^25^.

Oral administration reduces both glutathione and thiol levels in the intestinal homogenate more sharply than intranasal administration by the same dose, suggesting local intestinal oxidative stress. HCN may disrupt gut barrier integrity and interact with intestinal epithelial cells, depleting antioxidants.

The HO group elevated the insulin level compared to the control. But this elevation did not reach statistical significance, which may be due to the dosage of HNC used or the durations of exposure. The HO group has a slightly higher insulin level compared to the HI group, which may be due to the differences in absorption, bioavailability, and metabolism between the oral and intranasal routes. Further studies with different dosages of HNC and duration are needed. Also, toxicokinetic need to be studied to elucidate route-dependent toxic effects of HNC. X-ray imaging of halloysite treatment, either oral or intranasal, showed skeletal immaturity with reduced clarity of bone margins, incomplete vertebral column, hypoplastic skulls, and bilaterally distributed ribs. These effects suggested delayed ossification and developmental disruptions that may be due to the teratogenic effect of HNC. Nanomaterials like nano-TiO2 delay the fetal skeletal development by causing a reduction in calcium and zinc in the fetus and the maternal serum^26^.Other nanomaterials, like SiNPs, induce reactive oxygen species in zebrafish embryos^27^.

The study’s histopathological examination of rat lungs showed marked congestion, edema, hemorrhages, and bronchitis following both intranasal and oral halloysite treatment. These findings are consistent with Wang et al. ^12^, who observed a dose-dependent effect of orally administered HNTs in mice over 30 days. At a low dose (5 mg/kg), HNTs stimulated growth without lung toxicity. However, a high dose (50 mg/kg) inhibited growth and induced lung oxidative stress and inflammation. Notably, Wang et al. ^12^ reported that at the high dose, HNTs were absorbed from the gastrointestinal tract and accumulated in the lungs, with significant aluminum accumulation potentially contributing to pulmonary fibrosis.

Sawicka et al. ^25^ examined how HNTs at doses of 10–200 µg/mL affected A549 human alveolar carcinoma epithelial cells and BEAS-2B human bronchial epithelial cells in terms of their short-term (24–72 h) and long-term (7 days) cytotoxic impacts. Their observations of cell contraction, morphological and size alterations, cytoplasmic vacuolization, cell surface folding, peripheral nuclei placement, and even a rise in nuclei unquestionably validated the lethal impact of HNTs at low dosages (5 µg/mL and 25 µg/mL).

The data also indicate mild to moderate congestion and edema in the intestines of rats treated with halloysite, regardless of whether it was administered intranasally or orally. This observation is consistent with findings by Hu et al^24^., who, in their study of sub-chronic oral toxicity of HNTs in mice, found that high doses (50 mg/kg) caused aluminum and silicon accumulation, oxidative damage, and a significant inflammatory response in the small intestine, characterized by elevated nitric oxide synthase (iNOS) levels and cyclooxygenase-2 (COX-2), in addition to the organ damage that mediated by iNOS and inflammatory response.

Lysosomal inclusion is recognized as a foamy macrophage in alveoli in halloysite-treated rodents. There are about 50 xenobiotics known to cause phospholipidosis; antibiotics, antidepressants, antipsychotics, antimalarials, and antiarrhythmic medications are among them. A large number of these are cationic amphiphilic drugs (CADs), which share physical characteristics due to a hydrophilic cationic side chain in their chemical structure^28^. It has been demonstrated that cationic amphiphilic medications bind polar lipids via hydrophobic and electrostatic interactions, causing the development of drug-lipid complexes that are difficult for lysosomal enzymes to break down, aggregate, and are stored as lysosomal lamellar bodies^29^. Halloysite is hydrophilic and binds with α-lipoic acid by electrostatic force^30^. By the same mechanism, the halloysite may induce phospholipidosis. Renne et al^31^. reported that the foamy macrophage associated with the inhaled silica may be due to inhibition of phospholipid catabolism in the lung, leading to buildup of surfactant protein A and surfactant lipoproteins or functional impairment of alveolar macrophages.

The fetal pancreas of rats treated with halloysite intranasally showed more marked pathological changes than the orally treated one. The intranasal route allows halloysite to bypass the blood-brain barrier through the olfactory or trigeminal pathways and may enter systemic circulation more effectively, leading to higher fetal exposure. Drugs coated in halloysite clay nanotubes may be enabled to reach the brain effectively when administered intranasally as compared with orally^32^.

Unless there is a lack of study addressing placental transfer, the molecular weight of HNC (294.2 g/mol) suggests potential for interaction with the placenta, like other xenobiotics with a molecular weight (MW) less than 1,000.

Fluorescent polystyrene nanomaterials (40 to 500 nm in diameter) crossed the placental barrier in trophoblast culture. Also, these particles, when injected into pregnant mice, were visualized in different organs of the fetus^33^. Given that HNCs are within the diameter range (30–70 nm), it is plausible that HNCs could also pass through placental cells.

## Materials and methods

### Ethical approval

Dedication to ethics: The experiment was approved by the Institutional Animal Care and Use Committee of Beni-Suef University (BSU-IACUC; approval number: 025 − 017), all methods were carried out in accordance with the relevant guidelines and regulations of BSU-ICAUC, AVMA, and reported in accordance with ARRIVE guidelines in the study’s ethical application to BSU-IACUC.

### Chemicals

Halloysite nanoclay was obtained from the Sigma-Aldrich company. The product number is 685,445, and the CAS number is 1332-58-7. The product powder is white to yellow to beige with a pH of 4.5-7.0, 30–70 nanometers in diameter, and 1–3 microns in length. The substance was prepared freshly by dissolving it in distilled water, followed by sonication before each use.

### Characterization

#### High-resolution transmission electron microscope (HR-TEM)

The suspension of the compound powder was sonicated for 10 min using an ultrasonicator (Crest Ultrasonics Corp., New Jersey, USA). Then, a few drops were loaded on a coated copper grid and allowed to dry. Finally, the grid loaded with the sample was examined by HR-TEM (JEOL, JEM 2100, Tokyo, Japan).

### Animals

A total of thirty primiparous female Wistar rats, weighing 140–170 g, and fifteen male Wistar rats, weighing 190–220 g, were used in this experiment (The source of rats is the National Research Center’s laboratory animal farm in Giza, Egypt). The rats were kept in plastic cages with controlled temperatures between 21 and 23 °C, relative humidity maintained at 45% to 55%, and a light-dark cycle of 12 h each. They were provided with tap water and food ad libitum during both the adaptation and experimental periods.

### Experimental design

The female and male rats were randomly divided into three groups (*n* = 10 females, 5 males each). control group (C): female rats received distilled water orally; oral-treated halloysite group (HO): females received halloysite at 75 mg/Kg b.wt orally^12^, and intranasal-treated halloysite group (HI): female rats received the same dose via the nostril^34^. All groups administered treatments three days per week from day zero to the 19th day of gestation, confirmed by pregnancy detection, with gentle and careful handling practiced, avoiding abortion.

#### Pregnancy detection

A polygamous breeding system was implemented by placing one male with two females in plastic cages, from which the males were removed after pregnancy was confirmed through the examination of a vaginal smear, abdominal palpation, and body weight.

##### Examination of a vaginal smear

**Fig. 9:**
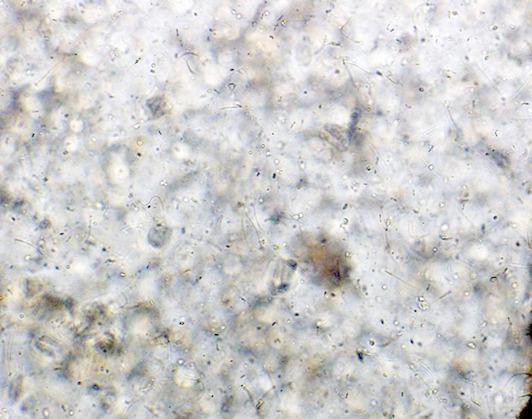
Microscopic examination of vaginal smear for sperm detection (10X).

Pregnancy detection was achieved by identifying the presence of sperm in the vaginal smear, a reliable indicator of copulation in rats. To rinse and aspirate the cells into the pipette, 200 µL of normal saline (0.9%) was aspirated using a sterilized disposable pipette. The posterior tip of the pipette was then placed close to the vaginal entry. A light microscope was employed to view the aspirated fluid at low power (10X) to observe sperm directly in the lavage cells (Fig. 9**)**, indicating whether mating had occurred or not^35^. The day on which sperm is detected in the vaginal smear is designated as the zero day of gestation.

##### Abdominal palpation

Additionally, by holding the rat gently from the back with one hand, it is possible to palpate the embryos through the anterior abdominal wall with the thumb and three fingers and confirm pregnancy between days 10 and 14^36^ to note if there is an abortion or not.

##### Body weight

Females’ body weight was recorded weekly as an indication of pregnancy^37^.

##### Euthanasia

On the 20th day of gestation, rats were given xylazine (8–12 mg/kg) and ketamine (90–120 mg/kg) intraperitoneally, blood samples were collected from the retro-orbital venous plexus by using hematocrit micro-capillary tubes without anticoagulant to obtain serum, then rats were humanely euthanized by cervical dislocation according to the AVMA Guideline for the Euthanasia of Animals. A thorough internal and external inspection was carried out, and any anomalies were noted. Pregnant female uteri and ovaries were inspected. Embryo weights were recorded. Then, lung and intestinal samples were harvested and kept at −80 °C for biochemical measures. In addition, embryos, lungs, and intestinal samples were harvested and preserved in 10% formalin for X-ray and histopathological examination.

### Biochemical measures

#### Total thiols

The method of Koster et al^38^. was adopted for the estimation of tissue total thiol content. Thiol content in tissue homogenate reacted with DTNB, forming a yellow-colored substance (2-nitro-5-thiobenzoate), and its absorbance was measured at 412 nm.

#### Glutathione (GSH) content

Glutathione content was determined using the method of Beutler et al^39^.with some modifications. The tissue homogenate was mixed with metaphosphoric acid to precipitate the proteins. GSH in the supernatant reduces DTNB to TNB⁻. Absorbance was read at 412 nm.

#### Lipid peroxidation

The MDA level was measured by centrifuging 250 µL of tissue homogenate for 10 min at 3000 rpm after precipitating it with 75 µL of 76% trichloroacetic acid. The obtained supernatant was combined with 175 µl of 1.07% thiobarbituric acid. Following a half-hour incubation period at 80 °C, 250 µL of 90% cold trichloroacetic acid was added. At 532 nanometers, the produced pink color was measured^40^.

#### Serum insulin levels

Serum insulin levels were measured by using an ELISA kit (Cat. No. 10-1113-01, Mercodia AB, Uppsala, Sweden)^41^.

### Anatomical examination

Skeleton examination: Fetuses were fixed in 90% ethanol solution for at least 2 weeks. After drying out, the fetuses were skinned completely. The internal organs were removed and skinned, and 0.5% potassium hydroxide (KOH) was then added. The KOH solution was drained out, the fetuses were rinsed using water, and the water was altered with a 1% solution of hydrogen peroxide once the purification was deemed to be complete. If the internal bones were white, the bleaching was excellent. Following a minimum of one week of immersion in 80% glycerol, the skeletal examination was carried out. The skull, spine, and ribs of the fetal skeleton were examined first, followed by the skeletons being turned over to view the anterior portions, which included the mouth cavity, the bones encircling the shoulders and hips, the forelimbs, and the rear limbs. Following the tests, the findings were documented, including the number of bones, position, morphology, and structure. Using X-ray imaging, the skeleton was inspected for additional assessment.

Soft tissue evaluation: The fetuses were kept in Bouin solution for one to two weeks before being dried and sliced in a specific manner. The forepart was cut and removed, the abdominal skin was sliced with a knife, and the organs within the abdominal cavity were gently removed.

### Histopathological examination

Embryos, dams’ lungs, and intestinal samples were collected and preserved in 10% formalin. Dehydrated in ethanol and cleared in xylol. Paraffin wax embedding, mounting onto a sliding/sledge microtome, sectioning in 4–6 μm thickness, hematoxylin and eosin (H&E) staining, and examination under a light optical microscope (Leica, Model 2500)^42^.

### Statistical analysis

The statistical program SPSS version 22 was used to examine all the data using one-way analysis of variance (One-way ANOVA), which was followed by a post hoc Tukey test. Data presented as mean and SE of mean and considered significant at *P-value* (*P* < 0.05).

## Conclusion

The halloysite nanoclay needs special attention for its use as a drug carrier, as it possesses toxic effects on dams and their fetuses. Halloysite causes toxicity to the dams, such as abortion, resorbed fetuses, and reduced litter size. In addition, it induced an evident oxidative stress. Histopathological findings revealed toxicity to pulmonary and intestinal tissue. The fetal pancreas of rats treated with halloysite intranasally showed more marked pathological changes than the orally treated one. The application of HNC during pregnancy should be handled with caution, particularly via intranasal installation.

### Limitations and recommendations

Limitations include the challenge of expecting long-term impacts and the possibility of interspecies diversity in transferring results to humans. Also, attention to developmental outcomes is among the recommendations.

Future studies ought to concentrate on the translocation of HNC across the placental barrier in ex vivo and in vivo animal models to accurately visualize and quantify HNC in fetal organs by using transmission electron microscopy and confocal laser scanning microscopy.

## Data Availability

All data generated or analyzed during this study are included in this published article.
